# Functional outcome and complication following THA through modified direct anterior approach correlated to cadaveric study: are there any differences in Asian hip?

**DOI:** 10.1186/s13018-021-02661-6

**Published:** 2021-08-20

**Authors:** Ong-art Phruetthiphat, Vasin Sangthumprateep, Songpol Trakulngernthai, Noppadol Aegakkatajit, Thanainit Chotanaphuti, Malee Chanpoo

**Affiliations:** 1 Orthopedic Department, Phramongkutklao Hospital, 315 Ratchvidhi Road, Thung Phaya Thai, Ratchathewee, Bangkok, 10400 Thailand; 2 Department of Anatomy, Phramongkutklao College of Medicine, Bangkok, Thailand

**Keywords:** Modified direct anterior approach, Lateral femoral cutaneous nerve of thigh, Cadaveric study, Clinical study, Functional outcome, Complication, Asian, Total hip arthroplasty

## Abstract

**Background:**

Direct anterior approach (DAA) has several advantages including shorter length of hospital stay, faster recovery, and better functional outcome while this approach may cause damage to the lateral femoral cutaneous nerve (LFCN) as high as 81% in the works of literature. Not much data has identified the LFCN pattern in the Asian population. Therefore, the purpose of our study was to identify characteristics of the LFCN patterns representing an Asian hip, which would aid to provide the most appropriate incision of modified direct anterior approach (MDAA) for total hip arthroplasty (THA), and to identify the clinical outcome and complications following THA through MDAA correlated with cadaveric hip in the Asian population.

**Methods:**

After IRB approval, a cadaveric study was done to identify pattern and course of the LFCN in Asian population. The MDAA defined as the incision 2 fingerbreadths posteriorly to anterior superior iliac spine to avoid injury to the LFCN. The clinical phase identified 32 patients who underwent THA because of late-stage osteoarthritis of the hip. The anterolateral skin numbness was measured along tensor fascia lata between 2 weeks until 2 years. The functional outcome assessed by Harris Hip Score (HHS) and complications were evaluated in all patients.

**Results:**

The characteristics of the LFCN from cadaveric study (phase 1) was predominantly in sartorius type (60.0%) followed by posterior type (26.6%), fan type (6.7%), and variant type (6.7%). The clinical phase demonstrated that 23 patients (71.9%) had no numbness while 9 patients (28.1%) came with numbness after undergoing THA through the MDAA. Finally, a small area of skin numbness remained in only 3 patients (9.4%) at 2 years follow-up. Additionally, there was no significant difference in functional score at 2 years follow-up (89.0 vs 91.2, *p* = 0.422) between those with LFCN injury and those without LFCN injury.

**Conclusions:**

The LFCNs were divided into four types. Modified direct anterior approach, which is an alternative approach for THA, allowing for a lower rate of skin numbness and faster recovery without hip dislocation, abductor weakness, and serious nerve complication. Functional outcome was comparable with and without LFCN injury.

**Level of evidence:**

Level II, prospective observation study

## Introduction

THA is one of the most successful procedures for severe OA hip to reduce pain, improve function, and increase the quality of life [1, 2]. Several approaches in THA can be utilized [3]. The standard posterolateral approach requires splitting of the posterior hip capsule and the external rotators, which is associated with high dislocation rates [4–7] while the anterolateral approach is more resistant to dislocation, but detaching the gluteus medius and minimus insertions from the greater trochanter are associated with abductor dysfunction and postoperative limp [3, 8, 9]. Direct anterior approach can be applied for several procedures on orthopedic surgery including femoroacetabular impingement or total hip arthroplasty for adult hip reconstruction [10–13], septic arthritis, or developmental hip dysplasia in pediatric orthopedic septic arthritis, or developmental hip dysplasia in pediatric orthopedic surgery [14–16] and irreducible closed reduction in displaced femoral neck fracture [17]. However, an anterior approach to the hip joint through an interval between sartorius and tensor fascia lata may cause damage to the lateral femoral cutaneous nerve (LFCN) as high as 81% in the literatures [18–20]. Recent publication has demonstrated three branching patterns and route of the LFCN from European specimens [21]. Conversely, not much data has identified the LFCN in the Asian population. Therefore, the purpose of our study was to identify characteristics of the LFCN patterns representing an Asian hip, which would aid to provide the most appropriate incision of modified direct anterior approach (MDAA) for total hip arthroplasty (THA), and to identify the clinical outcome and complications following THA through MDAA correlated with cadaveric hip in the Asian population.

## Materials and methods

### Phase 1: Cadaveric study

After Institutional Research Board Approval, 34 lateral femoral cutaneous nerve (LFCN) of thighs were done from 17 formalin-embalmed cadavers. We dissected the skin and subcutaneous tissue, in layers, from the abdomen to the thigh. The abdomen was opened, eviscerated, and the retroperitoneal fat was removed. We further identified the psoas muscle, lumbosacral plexus, especially LFCN structure and route. The lateral femoral cutaneous nerves of the thigh were carefully studied to identify their structure, boundary, branches, and nerve passage. Four cadavers were excluded because they were imperfect specimens. Finally, the study contained 30 specimens (24 specimens from male donors and 6 from female donors; mean age at death of 84 years; range, 76 to 94 years). All specimens were dissected following the dissection protocol through an ilioinguinal approach. The LFCN branching pattern and distribution within the proximal aspect of thigh were described with respect to 4 important landmarks; the ASIS, the inguinal ligament, the medial border of tensor fascia lata, and the lateral border of the sartorius.

**Fig. 1:**
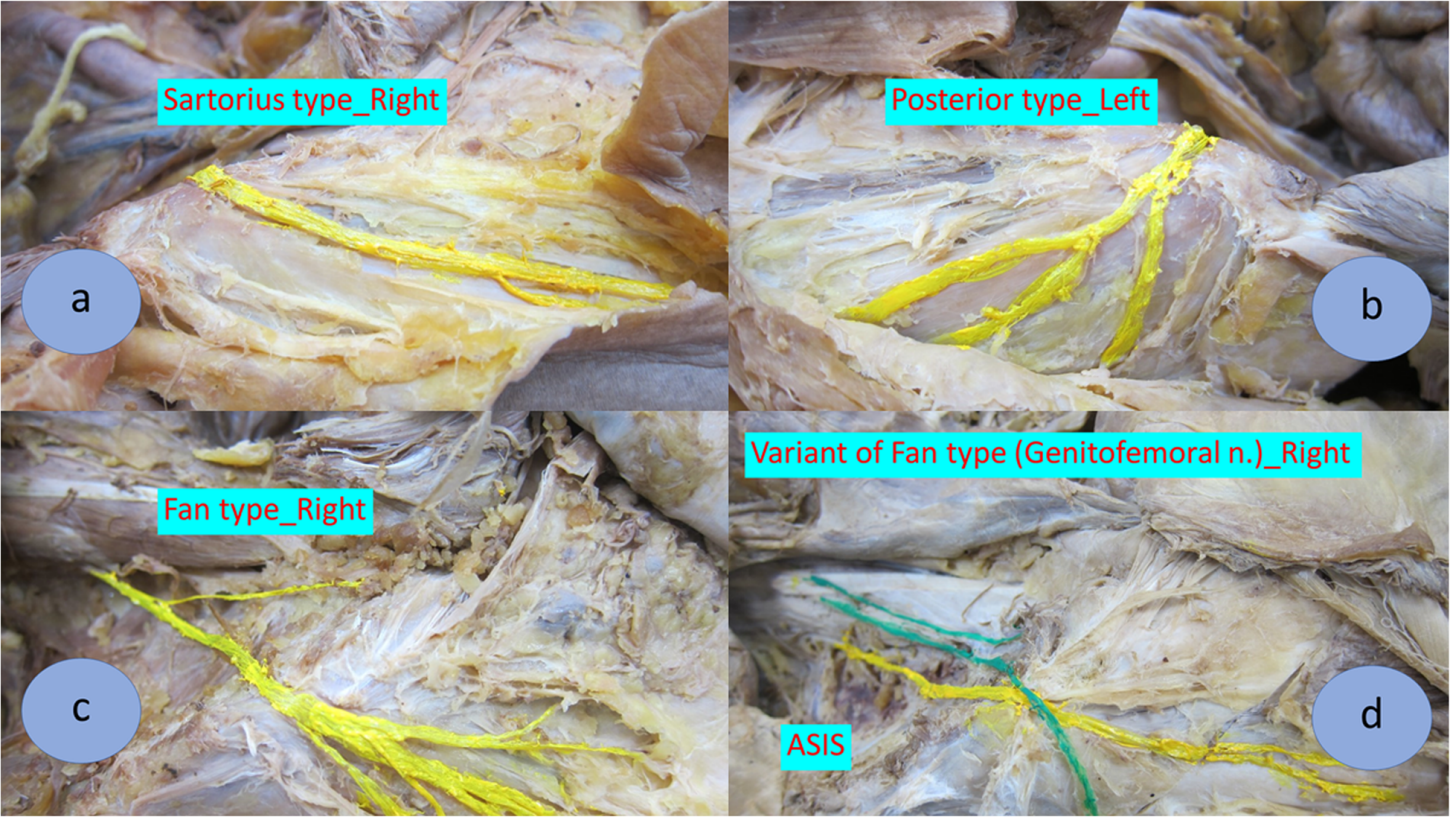
The pattern of lateral femoral cutaneous nerve of thigh (LFCN): (**a**) sartorius type, (**b**) posterior type, (**c**) fan type, and (**d**) variant type. ASIS is anterior superior iliac spine; Genitofemoral n. is genitofemoral nerve

**Fig. 2:**
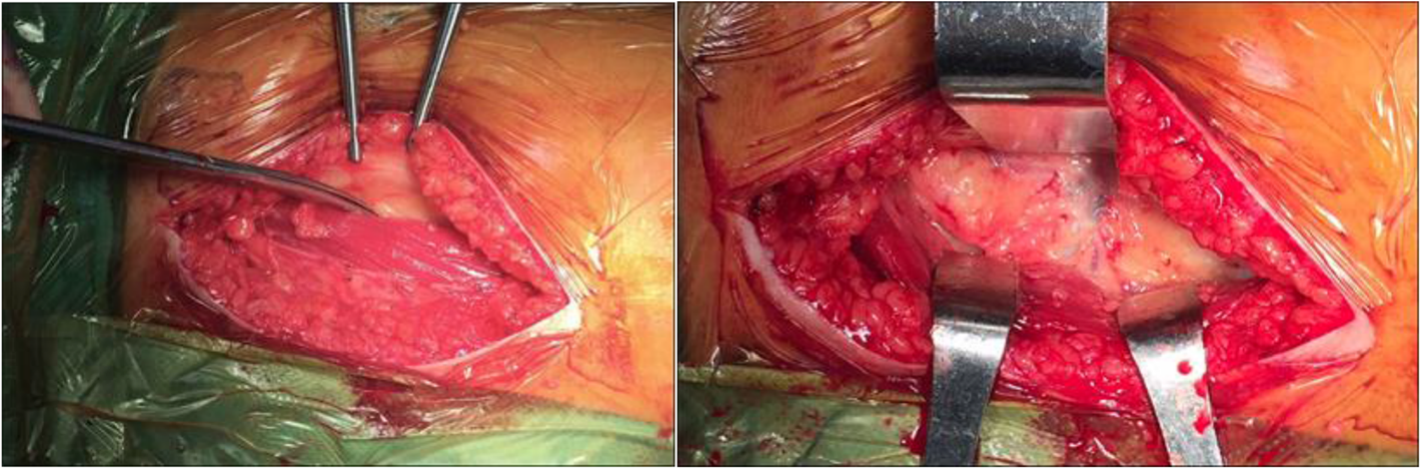
The modified direct anterior approach (MDAA): skin incision performed posteriorly to ASIS 2 fingerbreadths (left) and further dissection was deeply through the posterior one-third of tensor fascia lata (right)

The characteristics of the LFCN from cadaveric study (phase 1) was predominantly in sartorius type (60.0%) followed by posterior type (26.6%), fan type (6.7%), and variant type (6.7%) as shown in Fig. 1. The dominant branch of this nerve downwardly crossed along the lateral border of the sartorius muscle. Therefore, we further applied the most appropriate incision (the modified direct anterior approach; MDAA) for THA in 32 patients with late stage of osteoarthritic hip. The MDAA defined as the incision 2 fingerbreadths posteriorly to ASIS to avoid injury to the LFCN as shown in Fig. 2 (intraoperative picture).

### Phase 2: Clinical study

A prospectively observational study of 32 patients who underwent primary THAs for late-stage of osteoarthritis (OA) of the hip (26 patients from primary OA and 6 patients from secondary OA) of the hip between January 2016 and December 2017, was evaluated for areas of skin numbness and clinical outcomes following THA from a 2-week to a 2-year follow-up (FU). All patients had no clinical symptoms of lumbar nerve root pathology associated with lower spinal problem, no history of diabetic neuropathy, and no previous scar around the affected hip. Primary OA hip was defined as osteoarthritis of the hip which has no specific cause while secondary OA hip was defined as osteoarthritis of the hip with specific cause (osteonecrosis, posttraumatic, and developmental dysplasia of the hip). Patients’ variables reviewed were demographic data, comorbidity including American Society Anesthesiologist (ASA) classification and Charlson comorbidity index (CCI), preoperative visual analog scale (VAS), preoperative Harris hip score (HHS), intraoperative parameters including operative time (minutes), blood loss (milliliter), and intraoperative complication.

### Surgical procedure

All patients were performed on a single surgeon under spinal anesthesia. During an operation, all patients underwent a uniform surgical pattern, including a modified direct anterior approach skin incision, laying down on non-Hana surgical table, and a single prosthetic design (Pinnacle cup®, Marathon liner®, Metal head, Corail stem®, Johnson & Johnson, USA). To maximally avoid injury to the LFCN, all patients underwent the skin incision 2 fingerbreadths posteriorly to ASIS as shown in Fig. 2. The further dissection was deeply through the posterior one third of tensor fascia lata to avoid the major pattern (sartorius type) of the LFCN. We hypothesized that the application of the MDAA skin incision can avoid injury to the LFCN between 60% and 86.6% (60.0% from sartorius type and 26.6% from posterior type). However, we could not avoid injury to fan type (6.7%) and variant type (6.7%) to these nerves using this approach.

#### Measurement of numbness and follow-up

Area of anterior skin numbness was periodically evaluated at 6 weeks, 3 months, 6 months, 1 year, 1.5 year, and 2 years follow-up (FU). The measurement executed below the ASIS downwardly and the iliotibial band (anterolateral aspect of upper thigh) with a pin prick sensation until normal sensation occurred. We measured the skin numbness caused by LFCN injury, defined as an absence of normal sensation compared to the contralateral thigh. In addition, measurements of numbness performed by two physicians who were not involved in the operative field had averaged results.

#### Measurement of functional outcome and complication

Functional outcome following THA was measured by Harris hip score (HHS) at 2 years follow-up. The complication included paresthesia between numbness until meralgia paresthetica, abductor weakness (Trendelenburg gait), surgical site infection, and periprosthetic joint infection.

### Statistical analysis

Data description was based on means and standard deviation for continuous variables and absolute and relative frequencies for categorical variables. A standard Student’s *t* test was used for continuous variables while the Chi-squared test was applied for categorical variables. Statistical analysis was performed using STATA/MP 12 with statistical significance set to *P* < 0.05.

## Result

### Phase 1: Cadaveric study

The LFCN was identified in 30 cadavers. There were 24 men (80.0%) and 6 women (20.0%) with ages ranging from 76 to 94 years (average age, 84.4 years). These nerves were classified into 4 patterns (as shown in Table 1 and Fig. 1): sartorius type (60.0%, *n* = 18), posterior type (26.6%, *n* = 8), fan type (6.7%, *n* = 2), and variant type (6.7%, *n* = 2). The sartorius type defined as the main branch crossed along the lateral border of the sartorius muscle, the posterior type was characterized by a strong posterior nerve branch along the tensor fascia lata, the fan type was characterized by several spreading nerve branches with equal thickness, while the variant type defined as the combination of two nerves characterized by genitofemoral nerve joins with the LFCN.

**Table 1:** Demographic data of lateral femoral cutaneous nerves of thigh

Baseline characteristics	Type			
	Sartorius	Posterior	Fan	Variant
1. Site				
Right (*n* = 17) (56.7%)	11	4	1	1
Left (*n* = 13) (43.3%)	7	4	1	1
*p* value	0.918			
2. Gender				
Male (*n* = 24) (80%)	14	7	1	2
Female (*n* = 6) (20%)	4	1	1	0
*p* value	0.740			
Total (100%)	18 (60.0%)	8 (26.6%)	2 (6.7%)	2 (6.7%)

The anatomic distribution of the LFCN demonstrated correlation of these nerves with an important anatomical landmark as shown in Table 2. In most of the 30 dissections (63.3%, *n* = 19), the nerves cross overed the tensor fascia lata (TFL) while the others passed under the TFL (36.7%, *n* = 11). No significant difference was observed between the right and the left sides with respect to relationship among the nerve types (Chi-square test, *p* = 0.918) and there was no significant difference between gender (*p* = 0.740) and nerve patterns. All of the nerves crossed medially to the ASIS. Most of them branched out inferiorly to the inguinal ligament (90.0%, *n* = 27). According to the branches of the LFCN, almost of them (86.7%, *n* = 26) had at least 2 branches while only 4 dissections (13.3%) had no branch.

**Table 2:** Anatomic distribution of the lateral femoral cutaneous nerve (LFCN)

Characteristics	Number of specimens (*n* = 30)	Mean ± SD	Median (minimum-maximum)
1. Correlation of LFCN and TFL			
On TFL	19 (63.3%)		
Under TFL	11 (36.7%)		
2. Skin thickness (millimeter; mm)		3.6 ± 1.8	3.0 (2.0-8.0)
3. Correlation of LFCN and ASIS at inguinal ligament level			
Lateral to ASIS (mm)	0 (0.0%)	-	-
Medial to ASIS (mm)	30 (100.0%)	5.3 ± 7.1	3.0 (0.0-29.0)
4. LFCN divided into branches correlated with the inguinal ligament			
Divided proximally to inguinal ligament	3 (10%)		
Divided distally to inguinal ligament	27 (90%)		
5. Number of branches		2.6 ± 1.2	2.0 (1.0-6.0)
1	4 (13.3%)		
2	12 (40.0%)		
3	10 (33.3%)		
≥ 4	4 (13.3%)		
6. Size of nerve (the largest branch) (mm)		3.6 ± 1.3	4.0 (1.5-6.0)
7. Distance of nerve from ASIS to the nerve division (mm)		19.0 ± 15.4	15.5 (1.0-56.0)
8. Distance of nerve from ASIS to the most distal part (mm)		115.0 ± 32.0	114.0 (67.0-198.0)

### Phase 2: Clinical result

Patients’ demographics and comorbidities were demonstrated in Table 3. All patients underwent THA by using the modified direct anterior approach (MDAA, Fig. 2) to avoid an injury to this nerve. As a result (Table 4), 23 patients (71.9%) had no numbness after surgery while 9 patients (28.1%) came with numbness after undergoing THA through the MDAA. The average recovery of numbness was approximately 12.6 months (median 12.0 months). In those patients with numbness, 4/9 were fully recovered within 6 months and 2/9 were fully recovered at 1 year, while 3/9 were partially recovered at 2 years follow-up. Additionally, none of the patients had any surgical complications (Table 5) including neuropathic pain (meralgia paresthetica) and Trendelenburg gait, while one patient died from pneumonia with sepsis at 3 years after THA (3.1%, *n* = 1). Conversely, most of them (29 out of 32) (90.6%) had good to excellent functional outcome at 2 years follow-up (53.1% were excellent, 37.5% were good, and 9.4% were fair). Additionally, the pain score assessed by VAS (0.8 vs 8.2, *p* < 0.001) and the HHS (89.6 vs 42.8, *p* < 0.001) were significantly improved after THA through the MDAA (Table 5). Additionally, functional outcome evaluated by HHS (89.0 and 91.2, *p* = 0.422) and pain score assessed by VAS (0.8 vs 0.6, *p* = 0.314) were comparable between patients without LFCN injury and those with LFCN injury (Table 6).

**Table 3:** Patients demographics and comorbidities underwent THA through the MDAA

Parameters	Result (***n*** = 32)
Age^a^	61.2 ±14.5 (26-87)
Gender^b^	
Female	22 (68.8%)
Male	10 (31.2%)
Body weight (kg)^a^	63.3±16.9 (34-111)
Height (cm)^a^	160.0±5.0 (150-169)
BMI (kg/m^2^)^a^	24.7 ±6.3 (14.0-40.9)
Side for THA^b^	
Right	16 (50.0%)
Left	16 (50.0%)
Cause of osteoarthritis (OA) of the hip^b^	
Primary OA	26 (81.2%)
Secondary OA	6 (18.8%)
Length of stay (day)^a^	6.0 ± 1.9 (4-12)
Hypertension^b^	
Yes	17 (53.1%)
No	15 (46.9%)
Dyslipidemia^b^	
Yes	10 (31.3%)
No	22 (68.7%)
ASA class^b^	
1	5 (15.6%)
2	21 (65.6%)
3	6 (18.8%)
CCI^a^	2.8 ± 0.9 (2-5)

The modified direct anterior approach (MDAA); total hip arthroplasty (THA); American Society Anesthesiologist (ASA) classification; Charlson comorbidity index (CCI)

^a^Presented mean±SD (minimum-maximum)

^b^Presented *n* (%)

**Table 4:** Operative parameters, area of skin numbness (square centimeters), and their recovery time

Parameters	Primary THA through modified direct anterior approach(***n*** = 32)
**Operative parameters**	
Operative time (min)	105.1 ± 37.2 (50.0-200.0)
Blood loss (ml)	664.1 ± 340.1 (100.0-1450.0)
Skin incision*	11.8 ± 1.2 (9.0-15.0)
Numbness**^a^	
No	23 (71.9%)
Yes	9 (28.1%)
**Area of skin numbness (cm**^**2**^)*	The group with LFCN injury (*n* = 9)
6 weeks (*n* = 9)	234 ± 167.8 (56.3-600.0)
3 months (*n* = 9)	172.3 ± 127.9 (56.3-450.0)
6 months (*n* = 5)	140.0 ± 84.0 (25.0-200.0)
1 year (*n* = 5)	125.0 ± 43.3 (75.0-150.0)
1.5 year (*n* = 3)	96.0 ± 55.1 (40.0-150.0)
2 years (*n* = 3)	96.0 ± 55.1 (40.0-150.0)
**Recovery of numbness** (months)*	
Mean ±SD (min-max); median	12.6 ± 9.2 (3.0-24.0); 12.0
Rate of skin numbness**	
6 weeks	9 (28.1%)
3 months	9 (28.1%)
6 months	5 (15.6%)
1 year	3 (9.4%)
1.5 year	3 (9.4%)
2 years	3 (9.4%)

All of the 3 patients had approximately 40% remaining skin numbness at 2 years follow-up (FU) compared to 6 weeks FU

^a^Represented the prevalence of skin numbness occurred 28.1% (*n* = 9) from 32 patients that was consistent with our hypothesis

**Table 5:** Functional outcome and complications after THA through the MDAA in all patients

Parameters	Result (***n*** = 32)
**Harris hip score (HHS)**^**a**^	
Preoperative HHS	42.8 ± 14.8 (28.0-79.0)
Postoperative HHS	89.6 ± 7.0 (75.0-100.0)
*P* value	**< 0.001**
**Visual analog score (VAS)**^**a**^	
Preoperative VAS	8.2 ± 1.1 (6.0-10.0)
Postoperative VAS	0.8 ± 0.7 (0.0-2.0)
*P* value	**< 0.001**
Wound complication^b^	
Wound dehiscence	0 (0.0%)
Superficial infection	0 (0.0%)
Periprosthetic joint infection^b^	0 (0.0%)
Meralgia paresthetica^b^	0 (0.0%)
Abductor weakness^b^	0 (0.0%)
Venous thromboembolism^b^	0 (0.0%)
Mortality^b,c^	1 (3.1%)

The modified direct anterior approach (MDAA); Total hip arthroplasty (THA)

^a^Presented mean ± SD (minimum-maximum)

^b^Represented *n* (%)

^c^One patient died after 2 years follow-up (3 years after THA from pneumonia with sepsis)

**Table 6:** A comparison of functional outcome and pain score between those patients with LFCN injury and those patients without LFCN injury

Parameters	LFCN injury (*n* = 9)	Without LFCN injury (*n* = 23)	*P* value
Harris hip score (HHS)			
Pre HHS	41.7 ± 8.3	43.3 ± 14.1	0.754
Post HHS	91.2 ± 8.3	89.0 ± 6.6	0.422
HHS difference	49.6 ± 9.8	45.7 ± 14.4	0.467
Visual analog score (VAS)			
Pre-VAS	8.4 ± 1.1	8.1 ± 1.1	0.488
Post-VAS	0.6 ± 0.7	0.8 ± 0.7	0.314
VAS difference	7.9 ± 1.5	7.3 ± 1.2	0.248

## Discussion

Total hip arthroplasty can be performed in different approaches depending on the surgeon’s experience. Direct anterior approach (DAA) is currently a standard approach to avoid high dislocation rate as seen in standard posterolateral approach [4] and to avoid abductor weakness and postoperative limp from the anterolateral approach [10]. Additionally, the DAA has several advantages including shorter length of hospital stay, faster recovery, and better functional outcome [19, 22]. However, this approach may cause damage to the lateral femoral cutaneous nerve (LFCN) as high as 81% in the literatures [18–20]. Understanding the relevant anatomy is important before performing a surgical approach because the LFCN had several anatomical variations [21, 23–28] and not much data has identified the LFCN in the Asian population. Therefore, the purpose of our study was to identify characteristics of the LFCN patterns representing an Asian hip, which would aid to provide the most appropriate incision of modified direct anterior approach (MDAA) for total hip arthroplasty (THA), and to identify the clinical outcome and complications following THA through the MDAA correlated with cadaveric hip in the Asian population.

Rudin et al. demonstrated that LFCNs were divided into 3 types with almost equal distribution (36.0% in sartorius type, 32.0% in posterior type, and 32.0% in fan type) in European populations [21]. Another study by Sugano M et al. classified this nerve into 2 types (37.0% in anterior type and 63.0% in posterior type) and they did not identify the fan type pattern while 42.0% of all nerves crossed over the midline of tensor fascia lata [28]. Previous studies showed that injury to branches of LFCN cannot be avoided in almost one-half of cadaveric dissection. Our study showed the difference in the LFCNs pattern. The LFCNs were predominantly in sartorius type pattern (60.0%) and they were followed by posterior type (26.6%), fan type (6.7%), and variant type (6.7%). Even though small area of skin numbness remained in 3 patients (9.4%) at 1 year and 2 years follow-up, our cadaveric study possibly explained that fan type and variant type could not avoid the injury of LFCN (13.4%). Interestingly, this study showed the different nerve characteristics compared with the previous literatures [21, 24–28] and the clinical result after THA by the MDAA incision had a significantly lower rate of skin numbness (9.4%) compared to Yasuhiro H. et al.’ s study (31.9%). Therefore, an application of the MDAA can avoid injury to this nerve in clinical practice.

Previous literatures demonstrated superior functional outcome after an anterior approach compared to a posterior approach [22, 29–32]. Mark WZ et al. defined that THA through the direct anterior approach allowed shorter recovery time at 6 weeks (*p* < 0.0001) and length of stay (*p* < 0.0001) compared to the mini-incision posterior approach [22]. Yasuhiro et al. showed that those THA patients without LFCN injury had a higher quality of life (*p* = 0.01) assessed by Forgotten Joint Scale (FJS-12) than those THA patients with LFCN injury, while there was comparable functional outcome evaluated by WOMAC (*p* = 0.53) and Japanese Orthopaedic Association Hip Disease Evaluation Questionnaire (JHEQ) (*p* = 0.13) in both groups [33]. Additionally, Yu O. defined that there was similar functional outcome evaluated by WOMAC (*p* = 0.12) in the group without LFCN injury and the group with LFCN injury at an initial survey (1-year follow-up), but those with an LFCN injury with spontaneous nerve healing (2-year follow-up) were associated with a better quality of life assessed by WOMAC (*p* = 0.005), JHEQ (*p* = 0.003), and FJS-12 (*p* = 0.02) compared to those without spontaneous nerve healing [34]. Our study showed no significant difference in functional score at 2 years follow-up (89.0 vs 91.2, *p* = 0.422) between those with LFCN injury and those without LFCN injury that were similar to previous studies [33, 34].

This study firstly identified the combination of LFCN patterns from a cadaveric study and further adjusted the modified incision (the MDAA) to decrease the possibility of the LFCN injury during THA in a real clinical setting. This is additional information for clinical guidance for all orthopedists who performed THA through the direct anterior approach.

## Conclusion

Direct anterior approach causing damage to the lateral femoral cutaneous nerve (LFCN) is not uncommon. This study found that the LFCNs were divided into four types from cadaveric study. The modified direct anterior approach decreases the possibility of the LFCN injury, which is an alternative approach for THA allowing for a lower rate of skin numbness without hip dislocation, abductor weakness, and serious nerve complication. Functional outcome was comparable with and without LFCN injury.

## Data Availability

The datasets used and/or analyzed during the current study are available from the corresponding author on reasonable request.

## Abbreviations

LFCN: Lateral femoral cutaneous nerve of thigh
Modified DAA: Modified direct anterior approach
THA: Total hip arthroplasty
ASIS: Anterior superior iliac spine
TFL: Tensor fascia lata
OA: Osteoarthritis
ASA: American Society of Anesthesiologists classification
CCI: Charlson comorbidity index
VAS: Visual analog scale
HHS: Harris hip score
WOMAC: Western Ontario and McMaster Universities Arthritis Index
FJS: Forgotten joint scale
JHEQ: Japanese Orthopaedic Association Hip disease Evaluation Questionnaire
